# Worldwide Presence and Features of Flea-Borne *Rickettsia asembonensis*

**DOI:** 10.3389/fvets.2018.00334

**Published:** 2019-01-08

**Authors:** Alice N. Maina, Ju Jiang, Alison Luce-Fedrow, Heidi K. St. John, Christina M. Farris, Allen L. Richards

**Affiliations:** ^1^Viral and Rickettsial Diseases Department, Naval Medical Research Center, Silver Spring, MD, United States; ^2^Department of Biology, Shippensburg University, Shippensburg, PA, United States; ^3^Department of Preventative Medicine and Biostatistics, Uniformed Services University of the Health Sciences, Bethesda, MD, United States

**Keywords:** *Rickettsia*, *Rickettsia asembonensis*, flea-borne, worldwide distribution, arthropod hosts, *Rickettsia felis*-like organisms

## Abstract

*Rickettsia asembonensis*, the most well-characterized rickettsia of the *Rickettsia felis*-like organisms (RFLO), is relatively unknown within the vector-borne diseases research community. The agent was initially identified in peri-domestic fleas from Asembo, Kenya in an area in which *R. felis* was associated with fever patients. Local fleas collected from domestic animals and within homes were predominately infected with *R. asembonensis* with < 10% infected with *R. felis*. Since the identification of *R. asembonensis* in Kenya, it has been reported in other locations within Africa, Asia, the Middle East, Europe, North America, and South America. With the description of *R. asembonensis*-like genotypes across the globe, a need exists to isolate these *R. asembonensis* genotypes in cell culture, conduct microscopic, and biological analysis, as well as whole genome sequencing to ascertain whether they are the same species. Additionally, interest has been building on the potential of *R. asembonensis* in infecting vertebrate hosts including humans, non-human primates, dogs, and other animals. The current knowledge of the presence, prevalence, and distribution of *R. asembonensis* worldwide, as well as its arthropod hosts and potential as a pathogen are discussed in this manuscript.

## Introduction

*Rickettsia asembonensis* is a Gram negative, obligate intracellular bacteria of the order Rickettsiales and family Rickettsiaceae (1). Among *Rickettsia* spp. with validly published names, it is most closely related to *R. felis* (Table 1) (4–6, 8, 9, 11, 13–15, 17–19, 30). However, among incompletely characterize rickettsiae, *R. asembonensis* genetically groups with other *R. felis*-like organisms (RFLO). The RFLOs are genetically related to *R. felis* but consist of a unique group of rickettsiae that are associated with various arthropods including fleas, ticks, mites, and tsetse flies for which limited knowledge of their biology and pathogenicity is available (3, 16, 31). Unfortunately, the genetic information of the majority of RFLOs in the GenBank database is fragmentary. Of the RFLOs described, only *R. asembonensis* (32) and “*Candidatus* Rickettsia senegalensis” (3) have been cultured (from *C. felis*) and characterized.

**Table 1:** Worldwide distribution of *Rickettsia asembonensis* and closely related, incompletely characterized rickettsiae.

**Rickettsial agents**	**Source**	**Country**	**Location**	**Sequence comparison with R**. ***asembonensis*** **NMRCii (%)**						**Year in**	**References**
				**rrs**	**gltA**	**om pA**	**om pB**	**sca4**	**17kDa**	**GenBank**	
*Rickettsia felis* URRWXCal2	*Ctenocephalides felis*	USA	California. El Labs at Soquel	99.5	98	92.5	94.7	95.7	97.3	1999	(2)
“*Candidatus* Rickettsia senegalensis”	*Ctenocephalides felis*	Senegal	Dakar	99.4	98	-	94	94.7	-	2013	(3)
*Rickettsia* sp. RF2125	*Ctenocephalides canis*	Thailand	Sangkhlaburi District, Kanchanaburi province	–	99.3	–	99.7	–	–	2002	(4)
*Rickettsia* sp. cf1and5	*Ctenocephalides felis*	USA	Greenville County, South Carolina	–	99.5	–	–	–	100	2005	(5)
*Rickettsia* sp. SE313	*Echidnophaga gallinacea*	Egypt	Mansoura, Zagazig	–	99.7	–	–	–	100	2005	(6)
*Rickettsia* sp. cf9	*Ctenocephalides felis*	USA	Not provided	–	–	–	99.9	99.8	–	2006	Reeves et al., (Unpubl.)
*Rickettsia* sp. FS27	*Orchopeas horwadi*	USA	not provided	–	99.7	–	–	–	99.7	2006	Reeves et al., (Unpubl.)
*Rickettsia sp*.	*Ornithonyssus bacoti*	Egypt	Ebshaway, El Quseir, Qara Oasis, Zagazig, Arab El Maamal	–	–	–	–	–	100	#	(7)
Uncultured R. sp. Clone Hf56–2	*Archaeopsylla erinacei*	Germany	Bavaria	–	–	–	100	–	–	2008	(8)
Uncultured R. sp. Clone ARV5606	*Ctenocephalides felis*	Peru	Iquitos	–	99.7	–	–	–	99.7	2009	(9)
*Rickettsia* sp. RF2125	*Pulex irritans*	Hungary	Various parts of the country, specific information not provided	–	>99.3	–	–	–	–	#	(10)
*Rickettsia* sp. R14	*Ceratophylus fasciatus*	India	Not provided	–	99.7	–	99.9	–	–	2010	Chahota et al., (Unpubl.)
*R*. endosymbiont of *C. felis* isolate F143	*Ctenocephalides felis*	Thailand	No specific information provided (45 Districts)	–	99.7	–	–	–	100	2011	(11)
*R*. endosymbiont of *C. felis* isolate F144	*Ctenocephalides felis*	Thailand	No specific information provided (45 Districts)	–	99.7	–	–	–	100	2011	(11)
*Rickettsia* sp. clone 4-G/G/JP-10-2	*Ctenocephalides felis*	Costa Rica	Limon (Guacimo)	–	99	–	–	–	–	2011	(12)
*Rickettsia* sp. 'Synosternus'	*Synosternus pallidus*	Senegal	Dielmo	–	100	–	100	–	–	2011	(13)
*Rickettsia asembonensis* F30	*Ctenocephalides canis*	Kenya	Nyanza	100	100	99.8	99.9	100	100	2011	(14)
*Rickettsia asembonensis* F82	*Ctenocephalides felis*	Kenya	Nyanza	100	–	99.9	–	100	100	2011	(14)
Uncultured *R*. sp. Clone HL2a	*Ctenocephalides felis*	Malaysia	Kuala Lumpar, Selangor	–	99.7	–	–	–	–	2013	(15)
*Rickettsia* sp. RFLO-18	*Ctenocephalides felis*	Thailand	Was not deposited in the GenBank	–	–	–	99.7	–	–	#	(16)
*Rickettsia* sp. J28p	*Ctenocephalides felis*	Peru	Not provided	–	99.7	–	–	–	–	2015	Palacios-Salvatiera et al., (Unpubl.)
Rickettsial strain from *C. felis*	*Ctenocephalides felis*	Ecuador	Pastaza	99.9	99.7	–	100	100	100	#	(17)
*Rickettsia* sp. Clone Xr	*Xenopsylla ramesis*	Israel	Negev	100	99.7	100	100	–	100	2014	(18)
*Rickettsia* sp. 9AL	*Ctenocephalides felis*	Colombia	Villeta	100	100	–	100	–	–	2014	(19)
*Rickettsia sp*. 0095	*Macaca fascicularis*	Malaysia	Not provided	–	100	–	99.9	–	–	2014	(20)
*Rickettsia* sp. Clone Mal	*Homo sapiens*	Malaysia	University Malaya Medical Center	–	99	–	99.9	–	–	2015	(21)
Uncultured *Rickettsia* sp. Isolate F1	*Ctenocephalides felis*	South Africa	Mpumalanga Province	100	–	–	–	–	–	2015	(22)
*Rickettsia asembonensis* 0-TP-1	*Ctenocephalides felis*	Costa Rica	Cahuita, La Virgen, Limon, Tulialba, Guapiles	–	99.7	–	–	–	–	2016	(23)
*Rickettsia asembonensis* 6-CP-4-3	*Pulex simulans*	Costa Rica	Cahuita, La Virgen, Limon, Tulialba, Guapiles	–	99.7	–	–	–	–	2016	(23)
*Rickettsia asembonensis* 6-CP-4-4	*Ambylyomma ovale*	Costa Rica	Cahuita, La Virgen, Limon, Tulialba, Guapiles	–	99.7	–	–	–	–	2016	(23)
*Rickettsia asembonensis* CF26B/US	*Ctenocephalides felis*	USA	Orange County, California	99.9	99.7	99.9	99.9	100	–	2016	(24)
*Rickettsia asembonensis* Tapes	*Rhipicephalus sanguineus*	Brazil	Tapes	–	99.6	–	–	–	100	2016	(25)
*Rickettsia* sp. Clone SP003-M	*Ctenocephalides orientis*	Malaysia	Kuala Lumpur, Perak, Johore, Kelantan, Pahang, Negeri Sembilan	–	99.2	–	–	–	–	2016	(26)
*Rickettsia asembonensis* DB32B	*Rhipicephalus sanguineus*	Malaysia	Kuala Lumpur, Selangor, Pahang	–	99.6	–	–	–	–	2017	(27)
*Rickettsia asembonensis* CF#68	*Ctenocephalides felis*	Brazil	Maranhao State	–	99.6	–	99.9	–	100	2017	(28)
*Rickettsia asembonensis* F30	*Ctenocephalides felis*	Uganda	Southwestern Uganda	–	100	–	99.6–100	–	–	#	(29)
*Rickettsia asembonensis* 7.2	*Ctenocephalides felis*	USA	Galveston, Texas	–	–	–	–	–	100	2018	Quade et al., (Unpubl.)
*Rickettsia asembonensis* VGD7	*Ctenocephalides felis*	Peru	Peruvian Amazon	–	99.8	99.8	100	99.8	100	2017	(30)

*Rickettsia felis and Candidatus Rickettsia senegalensis are provided as reference rickettsiae that are closely related to but distinct from Rickettsia asembonensis*.

Other flea-borne rickettsiae include, besides the aforementioned *R. felis* and “*Ca*. R. senegalensis,” *Rickettsia typhi*, a member of the typhus group of rickettsiae (TGR). *R. typhi* is the causative agent of murine typhus, a febrile disease that is found throughout the world. *R. typhi* is vectored by various flea species-especially *X. cheopis*, but also other *Xenopsylla* species such as *X. astia* and *X. brazilliensis* (33, 34), *Synosternus pallidus*, and rarely, but importantly, *Ctenocephalides felis* the common cat flea that readily parasitizes cats, opossums, and other domestic, peri-domestic, and wild animals. *C. felis* is believed to be capable of hosting *R. typhi* and to vector murine typhus in areas outside the traditional range of rat fleas and rats (35, 36).

*R. felis, R. asembonensis*, and “*Ca*. R. senegalensis” fall within the spotted fever group rickettsiae (SFGR) that genetically clusters within the transitional group of rickettsiae (37). *R. felis* is associated with flea-borne spotted fever (38, 39) and the pathogenicity of *R. asembonensis* and “*Ca*. R. senegalensis” is currently unknown. These three agents have worldwide distribution, are often sympatric and most often found parasitizing cat and dog fleas (3, 4, 14, 38, 40, 41).

“*Candidatus* R. senegalensis” was first described in *C. felis* fleas from Senegal (3) and an agent believed to be “*Ca*. R. senegalensis”-like (*Rickettsia* sp. RF31) had been detected previously in *C. felis* near the Thailand-Myanmar border (4). A very close genetic relationship (99.9% based on *gltA* gene sequence) between *Rickettsia* sp. RF31 and the latter is notable (3). “*Ca*. R. senegalensis” is distinct from, but can be sympatric with, *R. felis* and *R. asembonensis* (40). It has worldwide distribution but is not reported as often as *R. felis* or *R. asembonensis*. Reports of its molecular presence in cat tissues suggests it may be able to infect vertebrate animals (41).

## History of *Rickettsia asembonensis*

**Figure 1:**
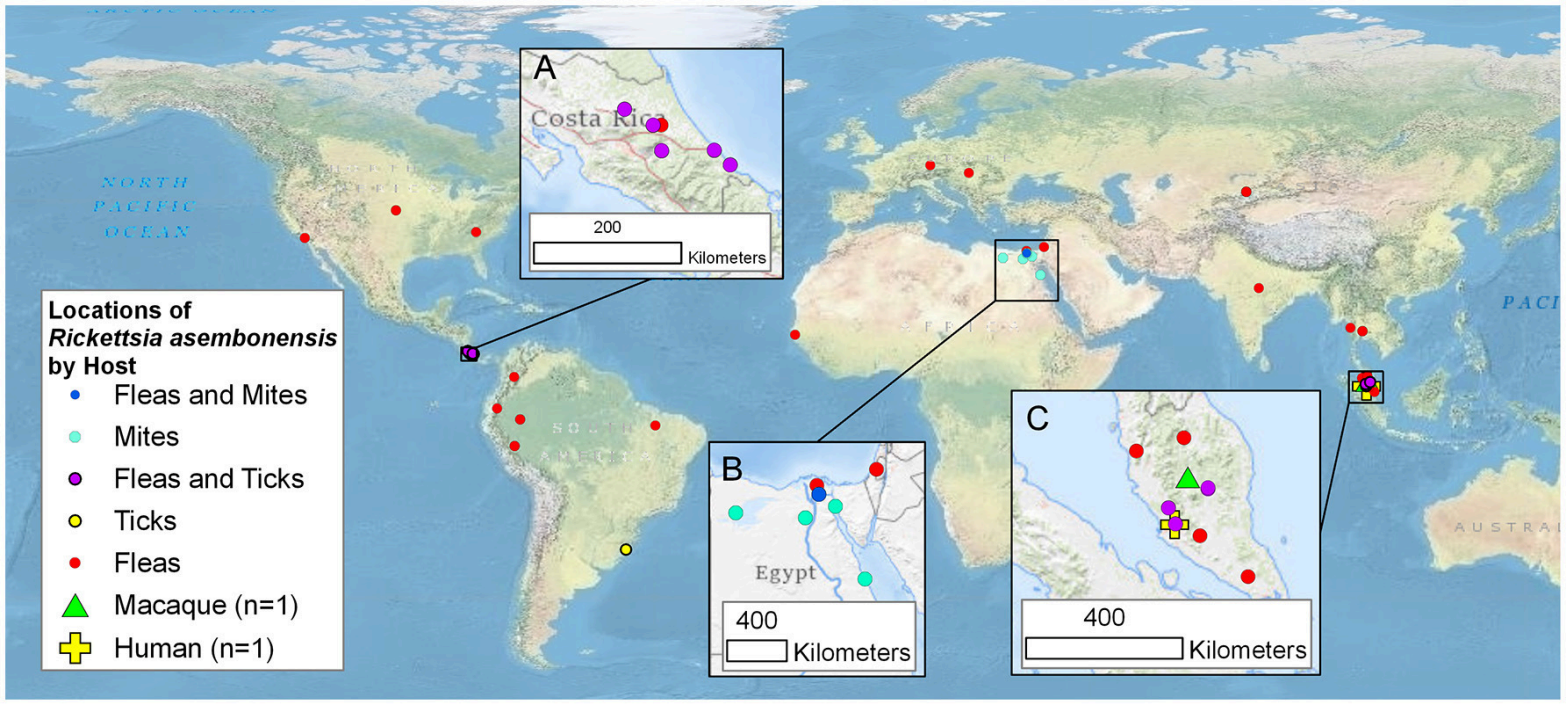
Worldwide mapof the locations of *Rickettsia asembonensis*, genetically similar rickettsiae, and associated vertebrate and invertebrate hosts. Inset maps are for points in **(A)**: Costa Rica; **(B)**: Egypt and Israel; and **(C)**: Malaysia. This map was created using ArcGIS® software by Esri. ArcGIS® and ArcMap™ which are the intellectual property of Esri and are used herein under license. Copyright © Esri.

Incompletely characterized rickettsiae with various identities most closely related to *R. asembonensis* populated the literature in the early 2000s (Table 1). These agents were detected by molecular techniques [i.e., PCR, nested PCR (nPCR), and/or quantitative real-time PCR (qPCR)] and then characterized by sequencing different size fragments of one or more commonly used gene targets (*rrs, gltA, ompA, ompB, sca4*, or the 17 kDa antigen gene). The first agent, referred to as *Rickettsia* sp. RF2125, was detected in *Ctenocephalides canis* in western Thailand near the Myanmar border (4). The agent was characterized by the sequence of a 1,171 bp fragment of the *gltA* that showed the rickettsial agent to be unique but most closely related to *R. felis* (4). The sequence of a 790 bp fragment of *ompB* (JX183538) from the original *Rickettsia* sp. RF2125 DNA preparation was obtained at that same time as the *gltA* but was not reported in the original article (4). It was reported in 2013 (14). We believe that RF2125 may have been the first detection of *R. asembonensis* or a very similar agent. Additional reports of *R. asembonensis* or an agent closely related to it continued to occur worldwide (Figure 1) shortly thereafter including: *Rickettsia* sp. cf1 and 5, USA (5); *Rickettsia* sp. SE313, Egypt (6); *Rickettsia* sp. Hf56-2, Germany (8); *Rickettsia* sp. ARV5606, Peru (9); and *Rickettsia* sp. Synosternus, Senegal (13). These partially characterized agents were described prior to our complete characterization of *R. asembonensis* (1). These agents are summarized along with *R. asembonensis* to include their distribution, vector hosts, and genetic characterization (see Table 1).

*R. asembonensis* was initially described as an unknown *Rickettsia* sp. detected in various flea species (i.e., *C. felis, C. canis, Echidnophaga gallinacean, X. cheopis*, and *Pulex irritans*) collected from various domestic animals (i.e., dogs, cats, and rodents) and houses (by light traps) in Asembo, Kisumu, in western Kenya during an epidemiologic surveillance study (14). This study was conducted concurrently with a fever study in which the presence of *R. felis* was identified in 7.2% of febrile patients (42). The initial molecular characterization of the *R. asembonensis* agent was accomplished utilizing a multilocus sequence typing (MLST) algorithm (43). Prevalence of this new agent (~91.7%) in collected fleas was found to be distinctly different from that of *R. felis* (8.3%) (14).

Subsequently, additional fleas collected from the same hosts and locations within the livestock-owning compounds in Asembo were processed for rickettsial culture. The new agent, *Rickettsia asembonensis* NMRCii, was successfully cultured from a pool of five individual flea triturate cultures isolated from *C. canis* and *C. felis* fleas obtained from domestic dogs. The cultures were initially grown in S2 and subsequently in C6/36 cell lines at 25°C (32), but not in Vero and L929 cell lines or embryonated chicken eggs incubated at 37°C (1).

The culture of *R. asembonensis* NMRCii was analyzed by microscopy, including Diff-Quik/acridine orange staining and transmission electron microscopy (32). The *R. asembonensis* were observed in the *Drosophila* S2 and *Aedes albopictus* C6/36 cells lines as early as 3 days post-infection, and could be observed at multiple time points throughout the average culture time of 40–45 days (32). Rickettsiae were observed both intra- and extracellularly at time points ranging from 15 to 30 days throughout the course of the continuous culture (32). The new agent was observed by acridine orange staining in singlets, doublets, and during heavy parasitization of host cells, in long chains (32). Transmission electron microscopy of the *R. asembonensis* revealed multiple free rickettsiae (round to elongated morphology) in the cytoplasm of the host cells, with normal rickettsial size [diameter 0.375–0.5 μm (round morphology), length 0.5–0.625 μm, width/diameter 0.25–0.375 μm (elongated morphology)]. A cell wall membrane, defined periplasmic space, and cytoplasmic membrane were observed, as well as the electron lucent “halo” (rickettsial slime layer) (32). Intranuclear localization/growth of the agent was not detected by acridine orange or by transmission electron microscopy (32).

Genetic characterization of the cultured *R. asembonensis* NMRCii by MLST using rickettsial genes *rrs, gltA, ompA, ompB*, and *sca4*; plasmid analysis; and whole genome sequencing confirmed that the new agent was indeed a unique *Rickettsia* species (1, 44). *R. asembonensis* NMRCii was shown to have an estimated genome size of 1.40 Mb, possessed a 21,692 bp circular plasmid and had a G+C content of 32.2%. The *R. asembonensis* plasmid, pRAS01, was discovered to be unique as it only shared 89% homology with that of *R. africae* ESF5 and only 84% homology with that of *R. felis*. The *R. asembonensis* genome has 1,147 predicted protein-coding genes, 33 tRNA genes, and three rrn operons. These characteristics are similar with those found within the genome of *R. felis* (NC_007109), which is 1.49 Mb in size and contains 1,400 protein-coding genes, 33 tRNA genes, and three rrn operons. Of the *R. felis* proteins, 1,157 (83%) have homologs in *R. asembonensis* (1, 44).

**Figure 2:**
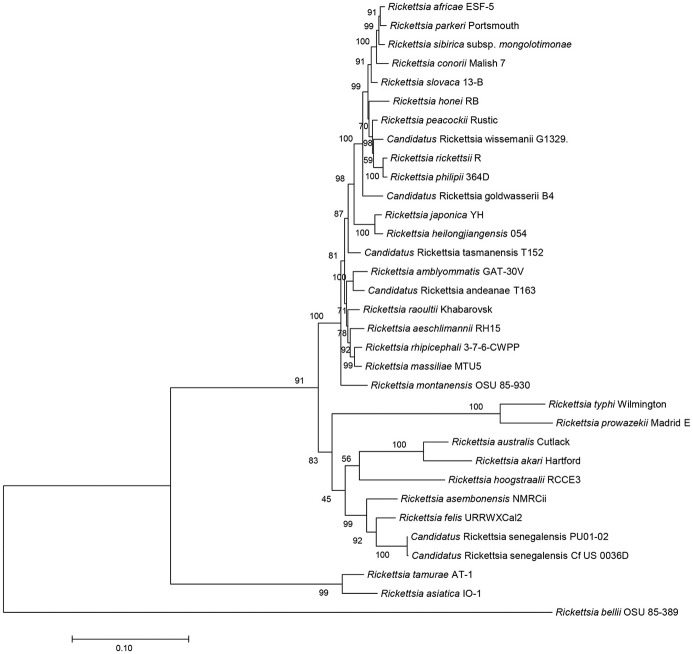
Molecular phylogenetic analysis using *ompB* open reading frame (4,130 bp). The evolutionary history was inferred by using the Maximum Likelihood method based on the Tamura-Nei model. The percentage of trees in which the associated taxa clustered together is shown next to the branches. Evolutionary analyses were conducted with MEGA7.

The sequences of *R. asembonensis* NMRCii, were 100% identical to those previously described for “*Ca*. R. asemboensis” isolates F30 and F82 for the following genes: *rrs, gltA, sca4*, and the 17kD antigen gene. For the *ompA* and *ompB* genes, the *R. asembonensis* NMRCii shared 99.86 and 99.98% similarity respectively, with the “*Ca*. R. asemboensis” isolates F30 and F82. The differences observed were as a result of nucleotide substitutions in two positions for the *ompA* gene and in one position for the *ompB* gene. A molecular phylogenetic analysis using 4,130 bp sequence of the variable gene-*ompB* open reading frame was conducted and the phylogenetic relationship between *Rickettsia asembonensis* NMRCii with *R. felis, Rickettsia* sp. PU01-02 (“*Ca*. R. senegalensis”) and other recognized *Rickettsia* species was determined (Figure 2).

*Rickettsia asembonensis* NMRCii was deposited in two separate culture collections (= DSM 100172^T^ and = CDC CRIRC RAS001^T^) and the name officially changed (according to the rules of the International Journal of Systematics and Evolutionary Biology) from “*Candidatus* Rickettsia asemboensis” to *Rickettsia asembonensis* (1).

## Arthropods Associated With *Rickettsia asembonensis*

*R. asembonensis* DNA has been detected in various arthropods, but most commonly in fleas (Table 1). It has been identified in fleas from three families namely the *Pulicidae, Ceratophyllidae* and *Coptopsyllidae*. In the cosmopolitan *Pulicidae* family it has been associated with seven genera: *Ctenocephalides* (*C. felis, C. canis*, and *C. orientis*); *Xenopsylla* (*X. cheopis, X. ramesis*, and *X. gerbilli*); *Archaeopsylla* (*A. erinacei*); *Echidnophaga* (*E. gallinacea*); *Pulex* (*P. irritans*); and *Synosternus* (*S. pallidus*). In the family *Ceratophyllidae, R. asembonensis* has been detected in three genera: *Ceratopsyllus* (*C. fasciatus); Orchopeas* (*O. howardi*); and *Nosopsyllus* (*N. laeviceps*) and in one genus in the family *Coptopsyllidae*: *Coptopsylla* (*C. lamellifer*) (45).

High prevalence rates of *R. asembonensis* have been reported in *C. felis* and *C. canis* (sympatric species), *S. pallidus, X. ramesis*, and *X. gerbilli* with up to 95, 95, 91.4, 100, and 33.3% of the fleas positive for *R. asembonensis*, respectively (13, 14, 18, 40, 46). Similar results in Costa Rica and Brazil confirm the high prevalence of *R. asembonensis* in *C. felis* (23, 28). In addition, *R. asembonensis* has been associated with other fleas, usually in much lower prevalence than in the aforementioned fleas. These include *E. gallinacea, P. irritans, C. lamellifer, X. hirtipes*, and *N. laeviceps*. Often these fleas are positive for *R. asembonensis* in the same areas as fleas highly infected with *R. asembonensis* (14, 46). The presence of the *R. asembonensis* in minimally infected flea species may be due to co-feeding and not that these fleas are reservoir hosts for *R. asembonensis*. Other arthropods in which evidence of *R. asembonensis* has been found include the tropical rat mites (*Ornithonysus bacoti*) in Egypt (7) and ticks (*Amblyomma ovale* and *Rhipicephalus sanguineus*) (23, 25–27).

## Pathogenicity

In limited laboratory studies no marked cytopathic effects were observed in S2 and C6/36 cells, beyond lysis of overly parasitized host cells (32). Additionally, no growth was observed in embyronated chicken eggs (1). Moreover, in two febrile studies conducted in Kenya no molecular evidence of this agent in patients' blood was seen whereas *R. felis* DNA was detected in 3.7 and 7.2% of fever patients' blood (42, 47). However, there is molecular evidence of *R. asembonensis* in a patient from Malaysia with fever, myalgia, arthralgia, mild headache, conjunctival suffusion, and the presence of petechiae noted on his limbs. Molecular analysis (*gltA* and *ompB* sequences) of the patient's blood identified *R*. sp. RF2125 (21). In addition, in the blood from a healthy free range domestic dog from Mnisi community situated in the northeastern corner of the Bushbuckridge Municipal Area, Mpumalanga Province, South Africa *R. asembonensis* was detected by NGS (22). Lastly, 12 of 50 healthy monkeys from Peninsular Malaysia had molecular evidence (100% *gltA* sequence similarity) of *R*. sp. RF2125/”*Ca*. R. asemboensis” (20). Thus, from the mixed results presented, the question of pathogenicity for humans and other animals is not yet resolved and requires more investigation.

## Future Research Direction

*R. asembonensis-*genotypes have been described in various biting and non-biting arthropods. Apart from *R. asembonensis* NMRCii that has been isolated in cell culture and whose full genome sequence is available in the GenBank Database, many of the others are just molecular isolates derived from arthropods with very limited sequence data for comparison. Functional and structural analysis of *R. asembonensis* is needed to ascertain differences and/or similarities between it and other rickettsial species. Moreover, research concerning the known/potential hosts of *R. asembonensis*, its current/potential arthropod vectors (both common and non-common), and its potential for interference with other rickettisal flea-borne pathogens (*R. felis* and *R. typhi*), as well as non-rickettsial pathogens such as *Yersinia pestis*, will be crucial to fully defining its pathogenicity and probability as a public health concern/nuisance across the world.

## Author Contributions

All authors contributed to the conception and design of the review. AM wrote the first draft of the manuscript. JJ, AL-F, HS, CF, and AR wrote revisions of the manuscript. All authors contributed to the manuscript's final version, and read and approved the submitted version.

### Conflict of Interest Statement

The authors declare that the research was conducted in the absence of any commercial or financial relationships that could be construed as a potential conflict of interest.
